# Barriers to health equity in the United States of America: can they be overcome?

**DOI:** 10.1186/s12939-025-02401-w

**Published:** 2025-02-07

**Authors:** Allen M. Chen

**Affiliations:** Department of Radiation Oncology, University of California, Irvine, Chao Family Comprehensive Cancer Center, 101 The City Drive, Building 23, Orange, CA 92868 USA

**Keywords:** Equity, Barriers, Health policy, Social determinants, Inclusion

## Abstract

Health equity—defined by the Centers for Disease Control and Prevention as " the state in which everyone has a fair and just opportunity to attain their highest level of health—” represents one of the most critical issues facing modern societies. While seemingly an increasing focus of policymakers in recent years, this concept is hardly a novel one. In 1948, the inaugural Constitution of the newly founded World Health Organization clearly stated that “the enjoyment of the highest attainable standard of health is one of the fundamental rights of every human being without distinction of race, religion, political belief, economic or social condition.” Yet nearly a century later, it is arguable how much progress society has made in achieving health equity, particularly in the United States of America where numerous factors at both the level of the individual and population contribute to significant complexity with respect to healthcare access and delivery. The purpose of this review is to thus outline the barriers to health equity so that thoughtful discourse can be promoted to create a more even playing field for the lives of the disadvantaged and underserved in the future.

## Introduction

According to the Centers for Disease Control and Prevention, health equity is “the state in which everyone has a fair and just opportunity to attain their highest level of health [1].” While seemingly an increasing focus of policymakers in recent years, this concept is hardly a novel one. In 1948, the inaugural Constitution of the newly founded World Health Organization clearly stated that “the enjoyment of the highest attainable standard of health is one of the fundamental rights of every human being without distinction of race, religion, political belief, economic or social condition [2].” Yet nearly a century later, it is arguable how much progress society has made in achieving health equity, particularly in the United States of America where notable imbalances exist across all levels of the population. Whether the issue is access, quality of care, or health outcomes, significant disparities continue to persist which underlie how many individuals view the healthcare system as a whole. From a practical standpoint, health equity is central to quality of care, profoundly affects the patient experience, and influence clinical outcomes across all diseases. However, impediments to health equity continue to be pervasive and need to be addressed so that society can move forward as a cohesive entity. The purpose of this review is to thus outline the barriers to health equity so that thoughtful discourse can be promoted to create a more even playing field for the lives of the disadvantaged and underserved in the future.

## Body

**Fig. 1:**
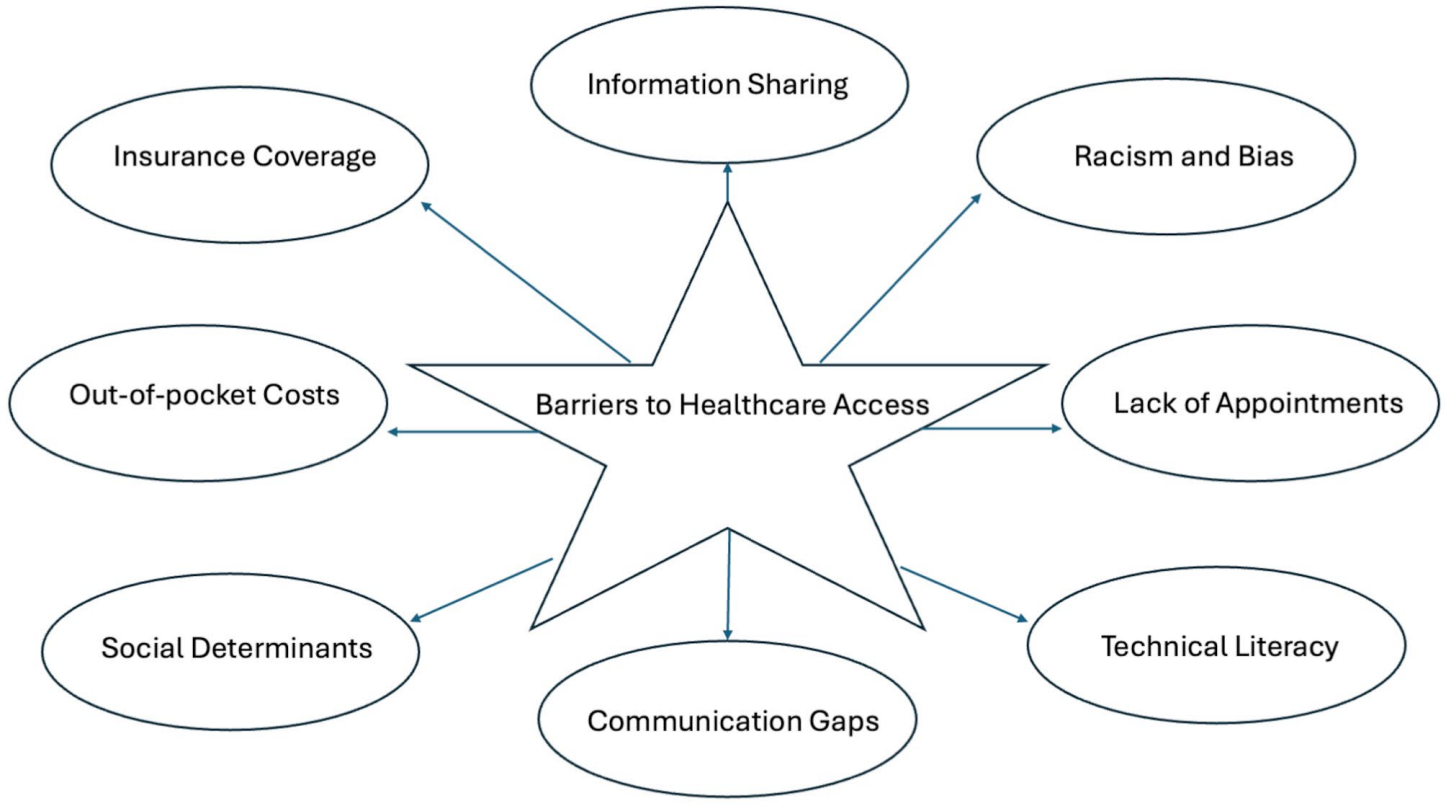
Barriers to access in healthcare

The barriers to health equity are multi-faceted, broad, and commonly overlapping (Fig. 1). While generally cited obstacles include those related to insurance coverage, affordability, social determinants, technical literacy and/or provider availability, among others, inequitable access to healthcare is a one of the most fundamentally pressing issues facing society. After all, how can citizens take a proactive approach to their own health if they feel rebuffed by a healthcare system they perceive as uninviting? Given the sheer volume of stakeholders operating in the healthcare marketplace, competing interests often mean that access, at least as defined by patients, is not prioritized or uneven across the population. The reasons underlying these inequities relate to an abundance of individual and systematic factors which will subsequently be discussed.

The question of who pays for healthcare can certainly influence health equity. Although the gains made with respect to the Affordable Care Act (ACA) to increase coverage have been well documented, a significant proportion of the public lack health insurance [3]. For others, just having insurance does not necessarily mean that access is guaranteed. For instance, studies have shown that prior authorization, which negatively impacts the ability of patients to see specialists, obtain diagnostic studies, acquire medications, and/or even start treatment can lead to major delays in care [4–7]. According to survey data of over 1,000 practicing physicians from the American Medical Association, more than half of respondents reported that prior authorization has “impacted patient job performance [7].” With respect to access, a staggering 94% of physicians stated that “delays in care” resulted from prior authorization. As concerningly, more than three-quarter of the physicians reported that “treatment abandonment” occurred because of prior authorization; and more than one-third reported that prior authorization led to a serious adverse event including hospitalization, disability, and/or death for a patient while waiting for care. Moreover, the negative effects of prior authorization on access can have a disproportionate impact on predominantly poor or disadvantaged communities [8–10]. For instance, hospitals and providers in underserved communities tend to operate on tight financial margins and have more limited resources, thus struggle with dedicating time to the laborious prior authorization process. One analysis found that prior authorization denials, in fact, were much more prevalent for underrepresented minorities, for those with limited education, and for those with low-income status [8]. Another study showed that minorities were less likely to receive the prescribed medication for diabetes due to prior authorization [9].

Even after their care has been approved by insurance, high out-of-pocket patient costs can pose another set of difficulties for many patients. When patients cannot afford medical care or find themselves choosing between medical care and paying for other basic obligations such as rent, mortgage, and/or food, they often go without healthcare. Indeed, the study of “financial toxicity” has gained increased attention given the disproportionately high amounts that many patients pay for healthcare compared to other services [11]. Data from West Health and Gallup poll found that 29% of adults reported putting off medical treatment because of out-of-pocket costs between 2001 and 2021 with low-income groups significantly more likely to skip or delay healthcare for a serious medical condition [12]. According to 2023 data from the Commonwealth Fund, the United States has the starkest income-based health disparities compared to other similarly developed nations [13]. Indeed, 46% and 27% of American adults skipped have skipped a medical visit, test, treatment, follow-up, or prescription fill within the last year solely because of cost among low-income and high-income earners, respectively.

Using data from 7.3 million health system visits, admissions, or prescriptions captured from various national registries between 2002 and 2016, Dieleman et al. showed that health care spending significantly varied by race and ethnicity across different types of care even after adjusting for age and health conditions [14]. Notably, White individuals were estimated to spend 15% more on ambulatory care than the rest of the population where Black individuals were estimated to spend and receive 26% less care. A more recent cross-sectional study of nearly 2 million Medicaid enrollees similarly showed that Black individuals generated lower spending and used fewer services, including primary care and recommended care for acute and chronic conditions than individuals from other backgrounds [15]. Although the reasons underlying these differences in healthcare utilization are likely multi-faceted, there is no question that cost still plays a role. For instance, recent data from the Commonwealth Fund continues to show that the percentage of Black individuals who avoided care in 2021 specifically because of cost was 11% and 18% in states with and without Medicaid expansion, respectively; for Latinos, the corresponding proportions were 16% and 23%, respectively [16]. Lastly, the implications of delaying and/or foregoing necessary care extend beyond worsening of disease. From a psychosocial standpoint, the inability to obtain timely care can lead to distress, anxiety, and further distrust of a health system that is too often criticized for being unfriendly. Additionally, delayed care can, quite paradoxically, lead to additional expense as complications from disease progression frequently necessitate more costly treatment.

**Fig. 2:**
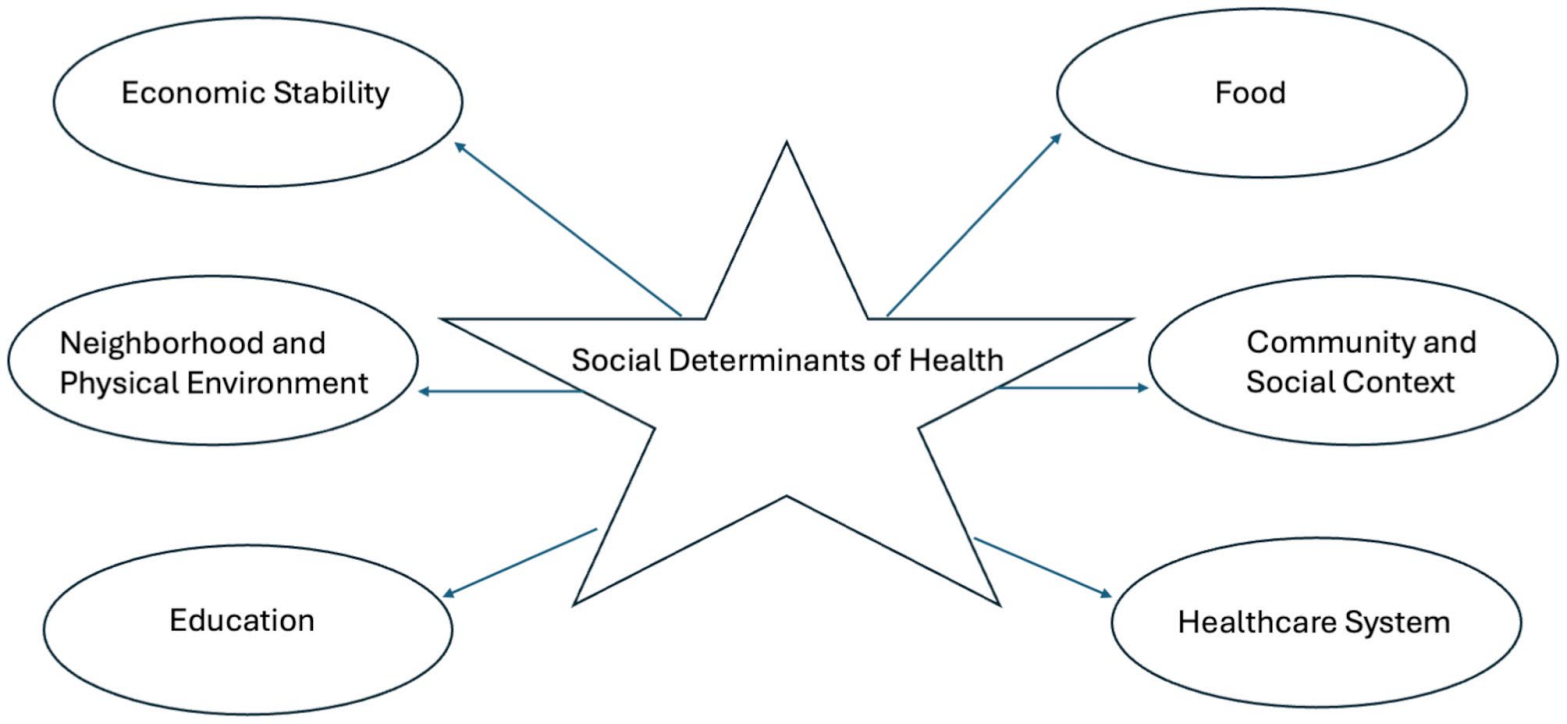
Social determinants of health

The influence of societal factors in creating inequities in healthcare has also been well established [17–19]. Social determinants of health (Fig. 2) including factors related to income, education, employment, housing, transportation, and geography, among others, have been shown to contribute significantly to health disparities and moreover, are often pervasive and deeply embedded across generations. For instance, studies have conclusively demonstrated that those who are born into poverty are more likely to stay in poverty [20]. Adler-Jackson et al. recently analyzed data from the United States Census and Health and Retirement Study to show significant associations between low socioeconomic status, Black race, and cognitive decline [20]. Indeed, the influence of economic stability, including an individual’s income and employment, on health has been consistently demonstrated. Similarly, education can provide individuals with the foundation they need to earn a higher income later in life, which allows them to access high-quality healthcare. As such, people with higher education have been shown to be healthier and to live longer [21]. Raghupathi et al. recently used empirical data from the Organization for Economic Development and Cooperation and the World Bank to demonstrate that adults with higher educational attainment have better health and lifespans compared to their less-educated peers [22]. Notably, the investigators concluded that tertiary education, particularly, is critical in influencing infant mortality, life expectancy, child vaccination, and enrollment rates—factors which have the potential to propel future generations forward on the socioeconomic scale.

Relatedly, a less-frequently mentioned determinant of health is the sense of control one has over life and work. Indeed, studies have increasingly shown that subjective social status—defined as how one perceives their position on the social hierarchy in relation to others— is an important predictor of health that affects the ability to fully participate in society and powerfully contributes to inequities across the population [23–26]. While higher social status typically confers a greater sense of control over one’s life and work, thus leading to better health; lower social status can engender feelings of disempowerment and humiliation, which can wear heavily on health. It is thus not surprising that the correlation between subjective social status and life expectancy is becoming increasingly recognized. In fact, arguments have been made that low social standing is seen not only as a condition of material deprivation but also as an indicator of an individual’s capability to control life and fully participate in society leading to psychosocial disadvantage and marginalization [23]. Demakakos et al. used Cox regression to model the associations between subjective social standing and and mortality in a sample of 9,972 adults from the English Longitudinal Study of Ageing over a 10-year period and demonstrated a strong association between social standing with all-cause, cardiovascular, cancer and other mortality [24]. In a subsequent analysis, the investigators showed a correlation between subjective social status and poorer outcomes using numerous health measures including self-rated health, long-standing illness, depression, hypertension, diabetes, central obesity, high-density lipoprotein cholesterol, triglycerides, fibrinogen, and C-reactive protein. Even after controlling for the potential influence of wealth, education, and occupational class, the effect of social standing was still evident, thereby suggesting that this classification influences health in a manner that is independent of financial means, resource availability and/or socioeconomic status. Similarly, Galvan et al. showed that economic circumstances and social status are distinct constructs that have distinct associations with health outcomes and well-being [26].

Along these lines, the importance of geography cannot be understated as higher-quality schools tend to be in more affluent neighborhoods. A person’s immediate surroundings also dictate lifestyle factors such as access to healthy foods, opportunities for physical activity, safe transportation, and other conditions such as water and air quality [27]. All of these variables can have a profound impact on health. For instance, Jones et al. conducted an analysis of 423 soil samples collected across various urban areas and showed that concentrations for toxic metals such as arsenic, cadmium, and lead were significantly elevated in low-income and predominantly minority communities [28]. The concept of “food deserts”— geographical regions, typically low-income in nature, where individuals lack access to high-quality, nutritious foods and/or grocery stores has been increasingly associated with health inequity [29]. Along similar lines, “physical activity deserts” have also been identified as neighborhoods where environmental circumstances preclude access to green spaces, parks, and recreational activities, all of which can promote healthy lifestyles [30].

People living in rural areas are also at risk for inequities in care due to specific obstacles related to the inability to travel into city centers or to take time off [31]. Patients have cited transportation and work-related concerns as a key limit on the ability to access preventive care and treatment. Indeed, statistics from the American Hospital Association estimate that approximately 3.5 million patients go without care because they cannot access transportation to their providers [32]. While shortages in healthcare services affect outcomes for individuals living in certain geographic neighborhoods, the root cause of health disparities are more deeply rooted than in just the lack of physical facilities.

The role of racism in contributing to health inequities must also be recognized. Stigma and bias have been well documented across the medical community, including discrimination based on race, immigration status, sex, gender, and sexual orientation. A position paper from the American College of Physicians (ACP) outlined how cultural factors hamper access to care and affect patients’ willingness and ability to seek specialized support such as mental healthcare services or pharmacologic therapy [33]. The authors highlighted the profound need for ending discrimination based on personal characteristics and ameliorating social determinants of health. More specifically, the ACP underscored the importance of increased efforts to address urgent public health threats, including injuries and deaths from firearms; environmental hazards; climate change; maternal mortality; substance use disorders; and the health risks associated with nicotine, tobacco use, and electronic nicotine delivery systems.

The relative lack of racial diversity in the healthcare workforce can also create a less than inviting environment for patients of different cultural backgrounds. This is particularly problematic given that nearly half of healthcare workers in the United States have witnessed racial discrimination against patients and say this is a crisis or major problem, according to 2024 survey by the Commonwealth Fund [34]. With respect to the workforce, nearly 6 in 10 Black healthcare workers and 4 in 10 Latino, Asian American and Pacific Islander workers say they have been discriminated against because of their race or ethnicity. According to findings from another large, nationally represented survey published by the Kaiser Family Foundation in 2023, the percentage of minorities who personally experienced discrimination in healthcare was frequent. In total, approximately 60% of Black adults, half of Native American and Latino adults, and 40% of Asian adults admitted to preparing for possible insults from providers or staff and/or felt they must be careful about their appearance to be treated fairly during health care visits. Furthermore, the survey found that patients who experienced discrimination were more likely to have reported feelings of anxiety, loneliness, and depression.

Structural factors also can contribute to barriers to access. For instance, decades-old policies like redlining (to designate “desirable” and “undesirable” neighborhoods) have led to both racial segregation and disparities in access to resources and services, like high-quality hospitals. The implications on healthcare access have been shown to be profound [35–37]. As one example, the Primary Care Development Corporation demonstrated that neighborhoods that were formerly redlined had a poverty rate nearly 4 times higher than of more “desirable” census tracts, and the proportion of Black people living in these areas was 9 times higher than in those A-rated census tracts [38]. Meanwhile, the rate of uninsurance—which can directly impact access to healthcare and healthcare affordability—was much higher in formerly redlined districts, with 18% of adults in those areas saying they don’t have payer coverage compared to 6% of those living in A-rated census tracts. A recent study confirmed the dramatically higher rates of chronic health conditions such as hypertension and diabetes in neighborhoods that were considered historically underserved [39].

As importantly, implicit bias can also instill distrust in medicine and dissuade patients of color from accessing care. Given that patients from disadvantaged backgrounds have reported more negative interactions with the healthcare system and are more likely to perceive their experiences as “cold, unfriendly, and insensitive,” factors related to comfort level can drive barriers to access [40]. In these cases, patients who feel that the healthcare system only caters to the privileged might fall through the cracks due to a perceived indifference for their beliefs and unique backgrounds. For instance, medical appointments can routinely conjure up emotions of fear and despair that can be exacerbated in certain underserved communities even accounting for intergenerational trauma. These negative feelings, which can compound the fear of having to navigate a complex and highly depersonalized healthcare system has been shown to be prevalent among the underrepresented [41]. Given that 1 in 5 households in the United States speaks a language other than English at home, the influence of cultural competency in creating access problems must be acknowledged [42].

While the digitization of healthcare has empowered some people, it is important to recognize that others are at risk for being left behind. Due to the pace at which technological innovation is changing healthcare, the lack of technical literacy for many patients, particularly those on the lower end of the socioeconomic spectrum, can also hinder activities such as scheduling appointments, checking results, and/or communicating with providers— tasks that are increasingly digitized in modern healthcare [43]. Indeed, underrepresented minorities have been shown to have more difficult accessing their medical records online [44]. More recently, the term “digital redlining” was introduced to describe racialized inequities in access to technology infrastructure, including access to health care, education, employment, and social services [45]. The importance of data equity in optimizing health outcomes for all is increasingly being recognized as well. For instance, the data which is collected and used to make evidence-based decisions in healthcare needs to be free of bias. Clinical trials, for instance, often enroll patients who are not reflective of the demographics from the general population [46–48]. Given a plethora of studies showing a disproportionate underrepresentation of minorities in research, the potential implications with respect to health equity cannot be understated.

While the barriers to health equity are numerous, efforts to address these are increasingly being proposed. For instance, there are a growing number of initiatives to address social determinants to promote health equity [49–51]. Central to these efforts is the resource deprivation theory which contends that the longstanding deprivation of resources experienced by underserved and/or vulnerable groups is central to health inequity [52]. While this theory advocates for the improved allocation of resources to address deficiencies, it must be recognized that resources are not restricted to material possessions, but include education, employment, housing, neighborhood safety, and psychological well-being, among others, which can be unevenly distributed to great degrees across society. In this sense, health initiatives that do not account for deeply rooted structural inequalities that the underserved face may do little to reduce chronically embedded disparity gaps. Regardless, the issue of how to prioritize resources to have the most meaningful impact on addressing health disparities remains uncertain.

Models under the Center for Medicare and Medicaid delivery system are increasingly addressing social needs and implementing community-based preventive programs [53]. Recently, numerous states required Medicaid managed care plans to screen for and/or provide referrals for social needs, and a recent survey found that nearly all responding plans reported activities to address social determinants of health [54]. To improve cultural literacy, the use of professional medical interpretation services and multilingual patient education materials can improve cultural responsiveness in healthcare [55]. With regard to access, educational initiatives are being explored to help patients understand the options for care delivery and the varying caliber of services available at different care facilities [56]. To reduce implicit bias in healthcare, programs to train staff in cultural competency and to create policies that are inclusive and sensitive to the needs of all have the potential to address longstanding disparities faced by disadvantaged groups [57]. Concerted efforts to create a healthcare workforce that is more reflective culturally of the general population are also being prioritized [58]. Ultimately, improving health equity will be critically dependent on promoting inclusivity at all levels such that a collective culture is created across society ensuring that nobody is left behind with respect to their ability to seek out the highest attainable level of health.

## Conclusion

The barriers to health equity are significant but not insurmountable. Successfully making progress will require a thoughtful, evidence-based approach with steady leadership and sustained engagement from a myriad of stakeholders with the goal of ultimately promoting high-quality care for all members of society. While a profound challenge, the journey toward bridging the many gaps is just beginning. Further research should focus on understanding the interaction among the many barriers to equity discussed and on identifying the areas that should be prioritized so that innovative solutions can be constructed. The need to develop and leverage partnerships between community-based organizations and the healthcare industry is also obvious. Indeed, how society chooses to define health equity in an ever-evolving landscape represents one of the foremost issues of the future.

## Data Availability

No datasets were generated or analysed during the current study.
